# Serum galactomannan antigen as a prognostic and diagnostic marker for invasive aspergillosis in heterogeneous medicine ICU patient population

**DOI:** 10.1371/journal.pone.0196196

**Published:** 2018-04-23

**Authors:** Yubhisha Dabas, Anant Mohan, Immaculata Xess

**Affiliations:** 1 Department of Microbiology, All India Institute of Medical Sciences, New Delhi, India; 2 Department of Pulmonary Medicine and Sleep Disorders, All India Institute of Medical Sciences, New Delhi, India; Azienda Ospedaliero Universitaria Careggi, ITALY

## Abstract

**Objective:**

This study was conducted to get a complete clinical and mycological picture of invasive aspergillosis (IA) in respiratory medicine ICU of a tertiary care hospital.

**Patients:**

From the cohort of 235 patients only one had proven IA. Based on AspICU algorithm, 21 had putative IA (8.9%), 12 were colonised (5.1%).

**Results:**

Adjusting the confounding factors, significant risk factors for IA were chronic obstructive pulmonary disease (COPD), temperature of ≥38°C, pneumonia and acute respiratory distress syndrome (ARDS). The best predictor of IA was AspICU algorithm (AUC, 1) followed by serum galactomannan antigen (GM) cut-off (≥1.24) calculated based on AspICU algorithm (AUC, 0.822). For 37% of patients, IA diagnoses was made earlier with serum GM than radiology. There were 70/235 (29.8%) deaths within 30 days of enrolment in the study. *Aspergillus* culture positivity (34/235, 14.5%) was associated with very high mortality (27/34, 79.4%), (p<0.05). The best predictor of mortality was GM cut-off (≥1.24) calculated based on AspICU algorithm (AUC, 0.835).

**Conclusion:**

This study imparts the focus on relatively underestimated *Aspergillus* infections prevalent in ICUs. The AspICU algorithm was found to be useful over others for IA diagnosis. The prognostic usefulness of serum GM antigen detection test highlighted overlooking the same may not be rewarding for the outcome of IA suspected ICU subpopulation.

## Introduction

Invasive Aspergillosis (IA) is considered a major threat for hemato-oncological and transplant patients whereas often underestimated in intensive care units (ICUs) [1, 2]. Existing literature suggests that the population at risk for IA has expanded to chronic obstructive pulmonary disease (COPD) patients, to the non-transplant intensive care patients; basically it now leans towards patients who do not present with the classical risk factors of IA [2–9]. The gold standard for IA definite diagnosis is the tissue histopathology which in critically ill patients is rarely established ante-mortem [1]. For IA diagnosis in neutropenic/transplant patient group, the European Organization for Research and Treatment of Cancer/National Institute of Allergy and Infectious Diseases Mycosis Study Group (EORTC/MSG) diagnostic criteria is followed which in many studies is used to extrapolate results in ICU settings as well [10, 11]. In recent years, another more feasible criterion specifically for critically ill patients has been introduced by Blot et. al. which clearly distinguishes Aspergillus colonisation from the infection cases [12]. This AspICU criterion is mainly preferable for ICU patients because of non-specific clinical signs and computerised tomography (CT) findings. Majority of the studies following this criterion are done retrospectively probably to simplify the interpretation of Aspergillus-positive lower respiratory tract samples [2, 12, 13].

In the medicine ICU of our hospital, we often encounter IA patients without any of the classical risk factors. Generally, the clinical manifestations in IA cases are pulmonary and lung biopsies aren’t feasible in this group of patients [2, 3]. The non-invasive serum or bronchoalveolar lavage (BAL) galactomannan antigen (GM) test still needs to be validated in this patient group. There is paucity of data available on the prognostic applicability of this diagnostic test in ICU settings.

The aim of this study was therefore to know the varied associated risk factors, evaluation of diagnostic algorithms, and role of galactomannan antigen assay in diagnosis and prognosis of IA in our medicine ICUs.

## Materials and methods

### Patient population and study settings

We conducted a cross-sectional study on the patients admitted in the medicine ICU of a tertiary care hospital with clinical suspicion of IA from August, 2012 to August, 2015. Patients who had any of the define host factors or microbiological evidence of *Aspergillus* infection during their ICU stay (with positive fungal culture or two positive circulating galactomannan tests) were included in the study. In addition, eligible patients could only be enrolled if they had at least two of the three following features: fever refractory to at least 3 days of appropriate antibiotics or fever relapsing after a period of defervescence of at least 48 hours while still receiving antibiotics; clinical signs and/or symptoms suggestive of invasive pulmonary mycosis: pleuritic chest pain or physical finding of pleural rub, or one of the following symptoms of lower respiratory tract infection (new sputum secretions, dypsnea, or hemoptysis); development of new pulmonary infiltrates on chest X-ray. However, chronic obstructive pulmonary disease (COPD) patients are the most common in ICU group with IA, fever can be present in a less fraction only (<40%) [14]. This could be due to administration of corticosteroids and IA being a relatively ‘quiet’ infection in immunological response terms which usually lyses fever. The sole exclusion criterion was clinical suspicion of IA before ICU admission.

This study was approved by the ethics committee of the institute i.e. All India Institute of Medical Sciences, New Delhi, India (Ref no. IESC/T-357/28.09.2012). The detailed procedure was as per institute guidelines: http://www.aiims.edu/aiims/academic/ethics-committee/forms%20in%20pdf/IEC/Format_of_Institution_Ethics_Committee_15032012.pdf. The consent forms for minor/incapable participants were obtained by their LAR i.e. legally accepted representatives (example: mother, father, children or grandparents).

### Classification of patients

Patients were classified according to AspICU algorithm [12] and EORTC/MSG diagnostic criteria [11]. However, EORTC/MSG criterion is not defined for ICU settings, the extrapolations of the same are common owing to the local heterogeneous ICU population [4, 15]. We extrapolated it to evaluate the usefulness of galactomannan Ag testing and it included addition of colonisation cases (repeat isolation of same fungus from a non-sterile site sample in absence of clinical features) and non-specific radiological signs (on chest X-ray/computed tomography (CT) with appearance of new signs of respiratory tract infection in patients for whom no alternative diagnosis was available). Radiological imaging was done for 165/235, 70.2% patients of which 132/165, 80% had positive but non-specific whereas 33/165, 20% had normal findings. The clinically suspected patients who did not comply with the diagnostic criteria definitions were categorised as no invasive aspergillosis (No IA). The clinical details of the patients with the 30 day mortality status to the day of admission were recorded. Only the patients with complete details were included in the study for uniform statistical analysis.

### Sample processing

All the samples were processed in class II biosafety hood. Total number and type of samples collected included 449 blood samples from 235 patients, 197 sputum samples from 188 patients, 41 bronchoalveolar lavage (BAL) samples from 24 patients, 39 endotracheal aspirate (ETA) samples from 23 patients and five pleural fluid samples from five patients. The samples were subjected to direct microscopy (KOH mounting and Grams staining) and growth on Sabouraud’s dextrose agar (SDA) (BD^™^ Difco^™^ 210940, USA) on different 25°C, 37°C and 45°C for up to five days of incubation [16, 17]. Sputum samples were mixed with an equal volume of 0.1% DL-dithiothreitol (DTT) and vortexed with sterile glass beads and incubated at 37°C for 15 min before processing further and BAL samples were semi quantitatively processed by the four-quadrant method to exclude any bacterial isolation [18, 19]. Quantitative cultures were also performed by serial dilution of the respiratory samples. The colony counts in colony forming units (CFU/mL) were calculated according to the visible number of colonies on SDA in relation to the dilution and inoculation factors [19] (data not shown).

### Galactomannan antigen

We performed the galactomannan antigen (GM) assay using Platelia kit (Bio-Rad, France). Serial serum samples (day 0: the day of enrolment of patient in the study; and day 7: the day after 7 days of the enrolment) were obtained for all the patients and 41 bronchoalveolar lavage (BAL) samples were collected from 24 patients. The results obtained were similar for both BAL and serum samples for the 24 patients. However, for a uniform GM analysis only the serum sample data is further discussed. According to the defined threshold in neutropenic patients, our threshold to define a positive GM was an optical density (OD) > 0.5. The first GM was the sample taken on the day of enrolment of the patient in the study. The delta GM (Δ GM) was the difference between the first GM and the second GM sample O.D.

### Statistical analysis

Socio-demographic, clinical characteristics and factors associated with development of IA were evaluated by univariate and multivariate analysis. For the simplicity of analysis; the proven case (one in this study, discussed in results) was merged with putative and probable IA in the AspICU algorithm and EORTC/MSG criteria classifications, respectively. The data collected was entered into a logistic regression model for calculation of unadjusted and adjusted odds ratio (ORs) described with 95% confidence intervals (CIs). A cut-off of P≤0.05, two tailed, was significant for all statistical analysis.

For determination of the best predictive cut-offs for IA diagnosis, receiver operating characteristics (ROC) curves were constructed for galactomannan antigen (GM). The relevant summary diagnostic parameters, including sensitivity, specificity, positive and negative predictive values (PPV, NPV), were calculated. ROC-curves also help us to evaluate a test for its overall discriminatory power and we have used them to evaluate the two diagnostic criteria and serum GM as markers for predictors of IA diagnosis and mortality. The areas under the curve (AUCs) were estimated to analyse the discriminatory power. Comparisons between AUCs were performed using the method of Hanley and McNeil [20]. All estimations were reported with 95% confidence intervals (CI). All statistical analysis were done using STATA version 9 (StataCorp. 2005. *Stata Statistical Software*: *Release 9*. College Station, TX: StataCorp LP).

## Results

From August, 2012 to August, 2015, there were total of 235 suspected cases of IA in the respiratory medicine ICU of our tertiary care hospital.

### Classification of patients

In the total cohort of 235, only one patient was categorised as proven case where *Aspergillus nidulans* was isolated repeatedly from blood samples; the patient developed metabolic encephalopathy and later multi-organ dysfunction and finally died of septic shock while receiving amphotericin B and voriconazole.

Based on the EORTC/MSG diagnostic criteria described in the study, we found 107 (45.5%) probable IA, 24 (10.2%) possible IA, 12 (5.1%) colonisation cases and 91 (38.7%) as No IA cases. Applying the AspICU algorithm, 21 had putative IA (8.9%), 12 were colonised (5.1%) and 201 were categorised as No IA cases (85.5%).

### Patient characteristics

The mean age for putative patients was (n = 22, mean ± SD = 49.18 ± 3.55, CI = 41.79–56.56) and for probable patients was (n = 108, mean ± SD = 51.78 ± 1.5, CI = 48.79–54.77). The characteristics of the patients with the independent risk factors are mentioned in Table 1.

**Table 1:** Statistically analysed different variables following the two diagnostic criteria.

Variables	Total	AspICU algorithm			p-value	OR (95% CI)	EORTC/MSG Criteria				p-value	OR (95% CI)
		Proven + Putative (n = 22; 9.4%)	No IA (n = 201; 85.5%)	Colonisation (n = 12; 5.1%)		Unadjusted	Proven+ Probable (n = 108; 45.9%)	Possible (n = 24; 10.2%)	Colonisation (n = 12; 5.1%)	No IA (n = 91; 38.7%)		Unadjusted
Host variables												
Haematological Malignancy	27	5 (18.5)	21 (77.7)	1 (3.7)	0.4	1.83 (0.68–4.94)	20 (74)	0	1 (3.7)	6 (22.2)	0.01	2.41 (0.93–6.24)
TB	216	18 (8.3)	187 (86.5)	11 (5)	0.18	0.43 (0.14–1.29)	93 (43)	24 (11.1)	11 (5)	88 (40.7)	0.01 F	0.27 (0.07–0.96)
COPD	41	19 (46.3)	18 (43.9)	4 (9.7)	0.00	21.25 (8.93–50.55)	29 (70.7)	1 (2.4)	4 (9.7)	7 (17)	0.2 F	3.7 (1.56–8.77)
ARF	15	8 (53.3)	7 (46.6)	0	0.00 F	8.52 (2.85–25.46)	9 (60)	3 (20)	0	3 (20)	0.43	2.66 (0.73–9.72)
HIV/AIDS	10	1 (10)	8 (80)	1 (10)	0.76	1.5 (0.3–7.42)	6 (60)	2 (20)	1 (10)	1 (10)	0.11 F	6 (0.74–48.17)
Diabetes	14	6 (42.8)	8 (57.1)	00	0.002 F	5.16 (1.66–16)	9 (64.2)	2 (14.2)	0	3 (21.4)	0.37 F	2.42 (0.65–8.94)
Mechanical Ventilation	95	19 (20)	74 (77.8)	2 (2.1)	0.00	2.77 (1.31–5.86)	65 (68.4)	11 (11.5)	2 (2.1)	17 (17.8)	0.00	5.14 (2.76–9.57)
Thrombocytopenia	85	8 (9.4)	71 (83.5)	6 (7)	0.59	1.28 (0.61–2.69)	41 (48.2)	5 (5.8)	6 (7)	33 (38.8)	0.3	0.99 (0.57–1.71)
Vasopressors	18	9 (50)	9 (50)	0	0.00 F	7.68 (2.78–21.16)	11 (61.1)	4 (22.2)	0	3 (16.6)	0.05 F	3.41 (0.95–12.13)
Immunocompromised	138	22 (15.9)	111 (80.4)	5 (3.6)	0.00	3.12 (1.3–7.51)	86 (62.3)	21 (15.2)	5 (3.6)	26 (18.8)	0.00	8.75 (4.79–15.95)
*H/o Corticosteroid administration*												
<7 days	47	5 (0.6)	39 (82.9)	3 (6.3)	0.84	1.27 (0.53–3.03)	26 (55.3)	4 (8.5)	3 (6.3)	14 (29.7)	0.44	1.63 (0.82–3.25)
> = 21 days	16	14 (87.5)	1 (6.2)	1 (6.2)	0.00	157.89 (19.76–1261.4)	14 (87.5)	1 (6.2)	1 (6.2)	0	0.001 F	
Antibiotic History	165	19 (11.5)	140 (84.8)	6 (3.6)	0.07	1.21 (0.53–2.74)	75 (45.4)	21 (12.7)	6 (3.6)	63 (38.1)	0.11	1.07 (0.6–1.91)
*Other Prolonged treatment*												
None	114	8 (7)	102 (89.4)	4 (3.5)	0.00 F	0.84 (0.69–1.04)	49 (42.9)	13 (11.4)	4 (3.5)	48 (42.1)	0.009	
ATT	33	1 (3)	31 (93.9)	1 (3)		0.54 (0.11–2.58)	17 (51.5)	2 (6)	1 (3)	13 (39.3)		1.11 (0.5–2.46)
HAART	3	0	3 (100)	0			2 (66.6)	0	0	1 (33.3)		1.45 (0.12–16.5)
Chemotherapy	8	1 (12.5)	7 (87.5)	0		1.21 (0.13–10.73)	8 (100)	0	0	0		
Others	51	0	48 (94.1)	3 (5.8)		0.53 (0.14–1.97)	15 (29.4)	7 (13.7)	3 (5.8)	26 (50.9)		0.69 (0.36–1.35)
*Duration In ICU (in days)*												
0–4	29	4 (13.7)	24 (82.7)	1 (3.4)		0.98 (0.64–1.48)	8 (27.5)	4 (13.7)	1 (3.4)	16 (55.1)		
>4–7	69	6 (8.7)	60 (86.9)	3 (4.3)		0.72 (0.21–2.36)	35 (50.7)	6 (8.7)	3 (4.3)	25 (36.2)		2.16 (0.89–5.22)
>7–14	104	9 (8.6)	89 (85.5)	6 (5.7)		0.8 (0.26–2.44)	49 (47.1)	11 (10.5)	6 (5.7)	38 (36.5)		2.13 (0.92–4.91)
>14	33	3 (9)	28 (84.8)	2 (6)		0.85 (0.22–3.32)	16 (48.4)	3 (9)	2 (6)	12 (36.3)		2.15 (0.77–5.96)
*Absolute Neutrophil Count (ANC) (/mm*^*3*^*)*												
<100	4	1 (25)	3 (75)	0	0.05 F	2.33 (0.2–24.2)	4 (100)	0	0	0	0.02 F	
100–500	135	10 (7.4)	114 (84.4)	11 (8.15)		1.28 (0.6–2.76)	55 (40.7)	11 (8.1)	11 (8.1)	58 (42.9)		0.69 (0.4–1.19)
>500	96	11 (11.4)	84 (87.5)	1 (1)			49 (51)	13 (13.5)	1 (1)	33 (34.3)		
Clinical variables												
*Temperature (°C)*												
<38	89	0	84 (95.7)	5 (5.6)	0.00 F	2.09 (1.12–3.91)	30 (33.7)	7. (7.8)	5 (5.6)	47 (52.8)	0.00 F	2.85 (1.64–4.93)
38–39.3	130	20 (15.3)	103 (79.2)	7 (5.3)		4.4 (1.62–11.9)	66 (50.7)	13 (10)	7 (5.3)	44 (33.8)		2.18 (1.25–3.8)
> = 39.4	16	2 (12.5)	14 (87.5)	0		2.4 (0.42–13.6)	12 (75)	4 (25)	0	0		
Pneumonia	80	13 (16.2)	66 (82.5)	1 (1.2)	0.00	1.43 (0.68–3.01)	60 (75)	13 (16.2)	1 (1.25)	6 (7.5)	0.00	14.97 (6.14–36.47)
Other Respiratory manifestations (ARDS etc.)	44	9 (20.4)	33 (75)	2 (4.5)	0.02	2.43 (1.08–5.47)	14 (87.5)	6 (13.6)	2 (4.5)	11 (25)	0.19	2.16 (1.03–4.53)
Sepsis	19	3 (15.7)	16 (84.2)	0	0.48 F	1.11 (0.3–4.06)	12 (63.2)	3 (15.7)	0	4 (21)	0.19	2.52 (0.81–7.87)
During ICU admission												
Neutropenia	35	6 (17.1)	28 (80)	1 (2.8)	0.2	1.6 (0.63–4.02)	22 (62.8)	6 (17.14)	1 (2.8)	6 (17.1)	0.02	3.57 (1.41–8.98)
Drug administration	6	0	6 (100)	0	1 F		3 (50)	0	0	3 (50)	1 F	0.62 (0.12–3.16)
Catheterisation	235	22 (9.4)	201 (85.5)	12 (5.1)			108 (45.9)	24 (10.2)	12 (5.1)	91 (38.7)	NA	
Malnourishment	10	10 (100)	0	0	0.00 F		10 (100)				0.008 F	
Mechanical ventilation	192	17 (18.4)	73 (79.3)	2 (2.17)	0.00	2.22 (1.06–4.63)	62 (67.3)	11 (11.9)	2 (2.1)	17 (18.4)	0.00	4.33 (2.54–8.79)
Dialysis	13	4 (30.7)	8 (61.5)	1 (7.6)	0.02	4.15 (1.27–13.58)	8 (61.5)		1 (7.6)	4 (30.7)	0.44 F	1.45 (0.43–4.85)
Corticosteroid administration	6	2 (33.3)	4 (66.6)	0	1.13 F	3.07 (0.54–17.5)	2 (33.3)	3 (50)		1 (16.6)	0.05 F	3.23 (0.37–28.16)
30 day mortality												
Survived	165	6 (3.6)	158 (95.7)	1 (0.6)	0.00	14.17 (5.7–34.75)	70 (42.4)	24 (14.5)	1 (0.61)	70 (42.4)	0.00	1.71 (0.94–3.12)
Expired	70	16 (22.8)	43 (61.4)	11 (15.7)			38 (54.2)	0	11 (15.7)	21 (30)		

Note: OR: odds ratio; CI: confidence interval; p-val: p-value; HM: haematological malignancy; TB: tuberculosis; COPD: chronic obstructive pulmonary disease; ARF: acute renal failure; HIV/AIDS: human immunodeficiency virus/acquired immunodeficiency syndrome; ATT: antitubercular treatment; HAART: highly active antiretroviral therapy; ARDS: acute respiratory distress syndrome; mech vent: mechanical ventilation

A total of 138 patients were immunocompromised (58.7%). The most common reason for ICU admission was respiratory distress (n = 124, 52.7%). The most common comorbid conditions were tuberculosis (n = 216, 91.9%), followed by chronic obstructive pulmonary disease (COPD) (n = 41, 17.4%) and hematological malignancies (n = 27, 11.5%). Ninety five (40.4%) patients were mechanically ventilated and eighteen (7.6%) were receiving vasopressors. Sixty three patients (26.8%) had received corticosteroids and sixteen (6.8%) of these patients were on prolonged (>21 days) corticosteroid therapy. Pulmonary manifestations (pneumonia and others including ARDS etc.) were diagnosed on admission in 124 (52.7%). Neutropenia was present in 139 patients (59.1%) and thrombocytopenia was seen in 85 patients (36.2%). On multivariate analysis, significant risk factors for the two diagnostic criteria are shown in Table 2.

**Table 2:** Multivariate analysis of risk factors for IA suspected cases defined according to AspICU and EORTC/MSG criteria.

	No. of patients	AspICU criteria Odd’s ratio (95% CI)	EORTC/MSG criteria Odd’s ratio (95% CI)
Haematological malignancy, n	27	0.42 (0.07–2.4)	1.59 (0.44–5.68)
Chronic obstructive pulmonary disease (COPD), n	41	9.96 (2.44–40.6)	1.92 (0.55–6.64)
H/o Corticosteroid administration, ≥21 days, n	16	83.6 (4.32–1614.5)	*NA
Antibiotic history, n	165	1.62 (0.39–6.62)	*NA
Absolute neutrophil count (ANC), <100 mm^3^, n	4	31.05 (0.89–1081.3)	*NA
ANC, 100–500 mm^3^, n	135	2.22 (0.64–7.63)	*NA
Temperature, 38–39.3 °C, n	130	3.07 (0.87–10.84)	2.24 (1.11–4.53)
Temperature, ≥39.4°C, n	16	3.69 (0.28–47.34)	*NA
Pneumonia, n	80	1.14 (0.22–5.98)	17.5 (5.42–56.46)
Acute respiratory distress syndrome (ARDS), n	44	1.96 (0.42–9.19)	4.27 (1.57–11.64)
Neutropenia during ICU admission, n	35	0.38 (0.06–2.37)	1.52 (0.42–5.41)
Mechanical ventilation during ICU admission, n	192	0.52 (0.11–2.46)	1.25 (0.42–3.63)
Dialysis during ICU admission, n	13	1.64 (0.2–13.2)	0.33 (0.05–2.08)
Steroid during ICU admission, n	6	0.06 (0.00–2.32)	0.9 (0.03–26.02)

Note:

*NA: not applicable; H/o: history of

Among the hematological malignancies the most common was acute myeloid leukaemia (AML) (7/27, 25.9%). There was only one patient with two underlying conditions (malignancy and tuberculosis). Three patients had underlying condition as metastatic cancers and cardiovascular manifestations each. There were 6 patients with liver manifestations. Only two patients had documented dissemination of intravascular coagulation (data not shown).

### Radiological findings

Chest X-ray or computed tomography (CT) was done for 165/235, 70.2% cases, of which 132/165, 80% had positive findings whereas 33/165, 20% had normal findings. Overall, it could not be done for 70/235, 29.8% patients.

The varied imaging (CXR/CT) findings are reported in Table 3. Using the fisher’s exact test, the *p* value was significant following both the diagnostic criteria (*p*<0.001).

**Table 3:** Radiological findings of the patients according to the two diagnostic criteria.

Radiological findings†	No. of patients	AspICU algorithm (p<0.001)			EORTC/MSG Criteria (p<0.001)			
		Proven+ Putative (n = 22; 9.4%)	No IA (n = 201; 85.5%)	Colonisation (n = 12; 5.1%)	Proven+ Probable (n = 108; 45.9%)	Possible (n = 24; 10.2%)	Colonisation (n = 12; 5.1%)	No IA (n = 91; 38.7%)
Not done, n (%)	70 (29.8)	0	59 (84.3)	11 (15.2)	0	0	11 (15.2)	59 (84.3)
Normal, n (%)	33 (14)	0	32 (96.9)	1 (3)	0	0	1 (3)	32 (96.9)
Pleural effusion, n (%)	10 (4.3)	0	10 (100)	0	9 (90)	1 (10)	0	0
Consolidation, n (%)	10 (4.3)	4 (40)	6 (60)	0	9 (90)	1 (10)	0	0
*GGO, n (%)	18 (7.7)	3 (16.6)	15 (83.3)	0	12 (66.6)	6 (33.3)	0	0
*GGO with consolidation, n (%)	71 (30.2)	12 (16.9)	59 (83.1)	0	62 (87.3)	9 (12.6)	0	0
Other combinations/ other findings, n (%)	23 (9.8)	3 (13)	20 (86.9)	0	16 (69.5)	7 (30.4)	0	0

Note:

*GGO: ground glass opacity;

^†^ these radiological findings include both chest X-ray and CT scan

The unadjusted odds ratio (95% confidence interval) were >1; for consolidation 5.05 (1.24–20.5), ground glass opacity (GGO) 1.51 (0.38–6.01), GGO with consolidation 1.54 (0.64–3.66) and other combinations/ findings 1.13 (0.29–4.4) whereas on multivariate analysis with all the confounding factors present no significance was noted (adjusted ORs <1).

### Galactomannan antigen testing

**Fig 1:**
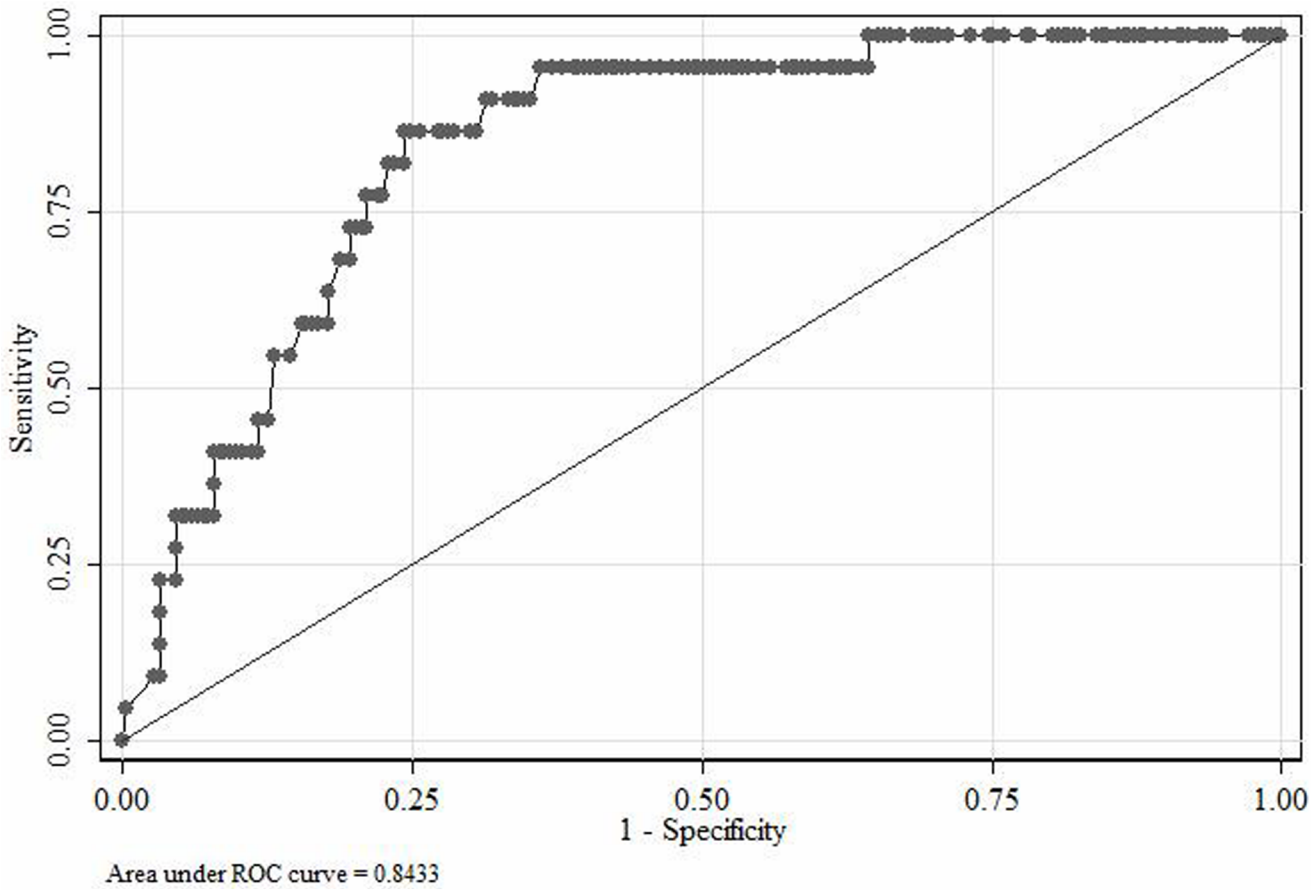
Significant GM cut-off on the basis of AspICU algorithm (for calculation proven and putative IA cases are taken as one group whilst combining others together).

Serial serum GM (day 0 and 7) was measured in all (n = 235) patients. The significant GM cut-off was ≥1.24 when calculated as per the AspICU algorithm with an AUC, sensitivity, specificity, negative predicted value (NPV) and positive predicted values (PPV) of 0.843, 77.27, 77.46, 97.06 and 26.15, respectively (Fig 1).

**Fig 2:**
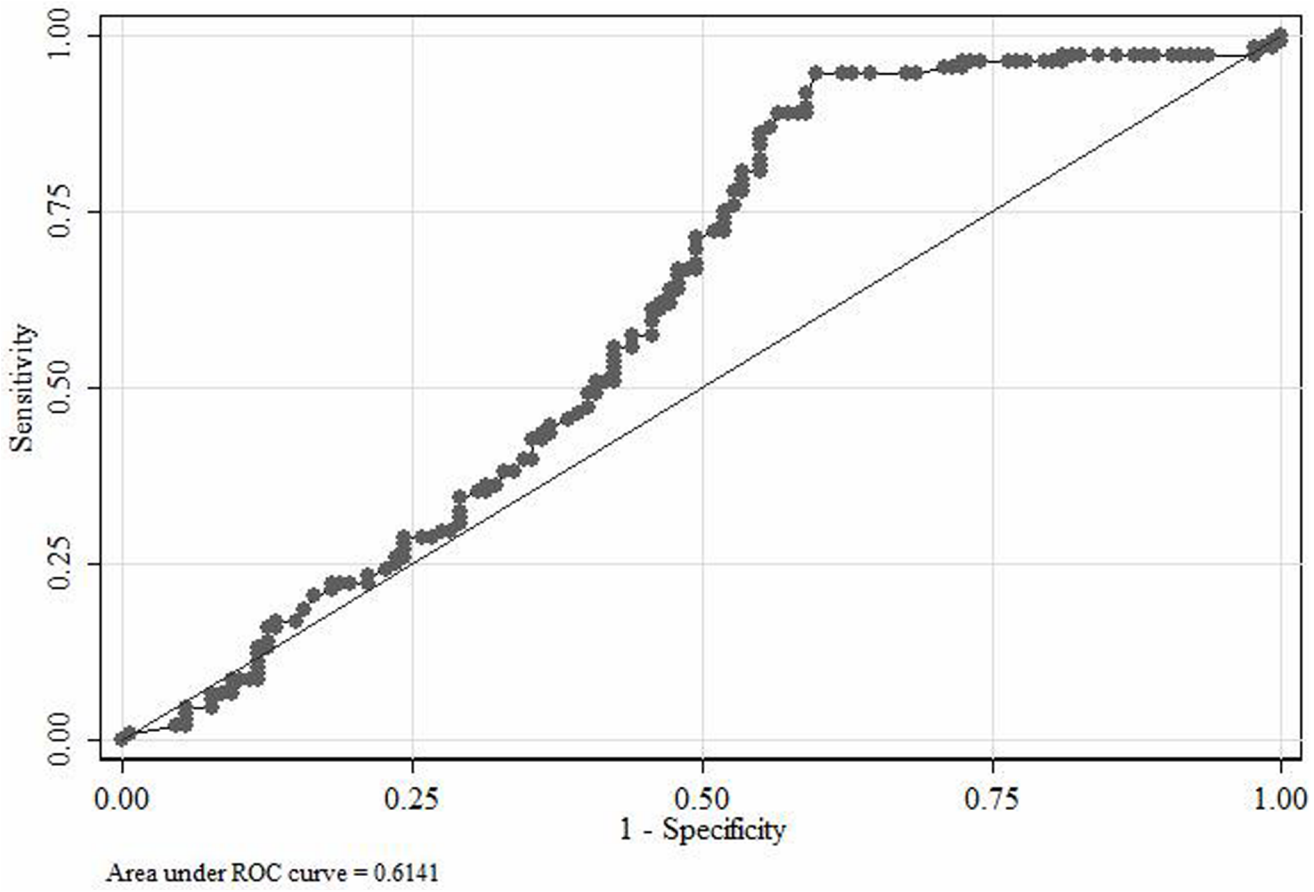
Significant GM cut-off according to IA cases defined on the basis of EORTC/MSG criteria (for calculation proven and probable IA cases are taken as one group whilst combining others together).

The significant GM cut-off was ≥0.78 when calculated as per the EORTC/MSG criteria with the AUC, sensitivity, specificity, NPV and PPV of 0.614, 57.41, 54.33, 60 and 51.67, respectively (Fig 2).

**Fig 3:**
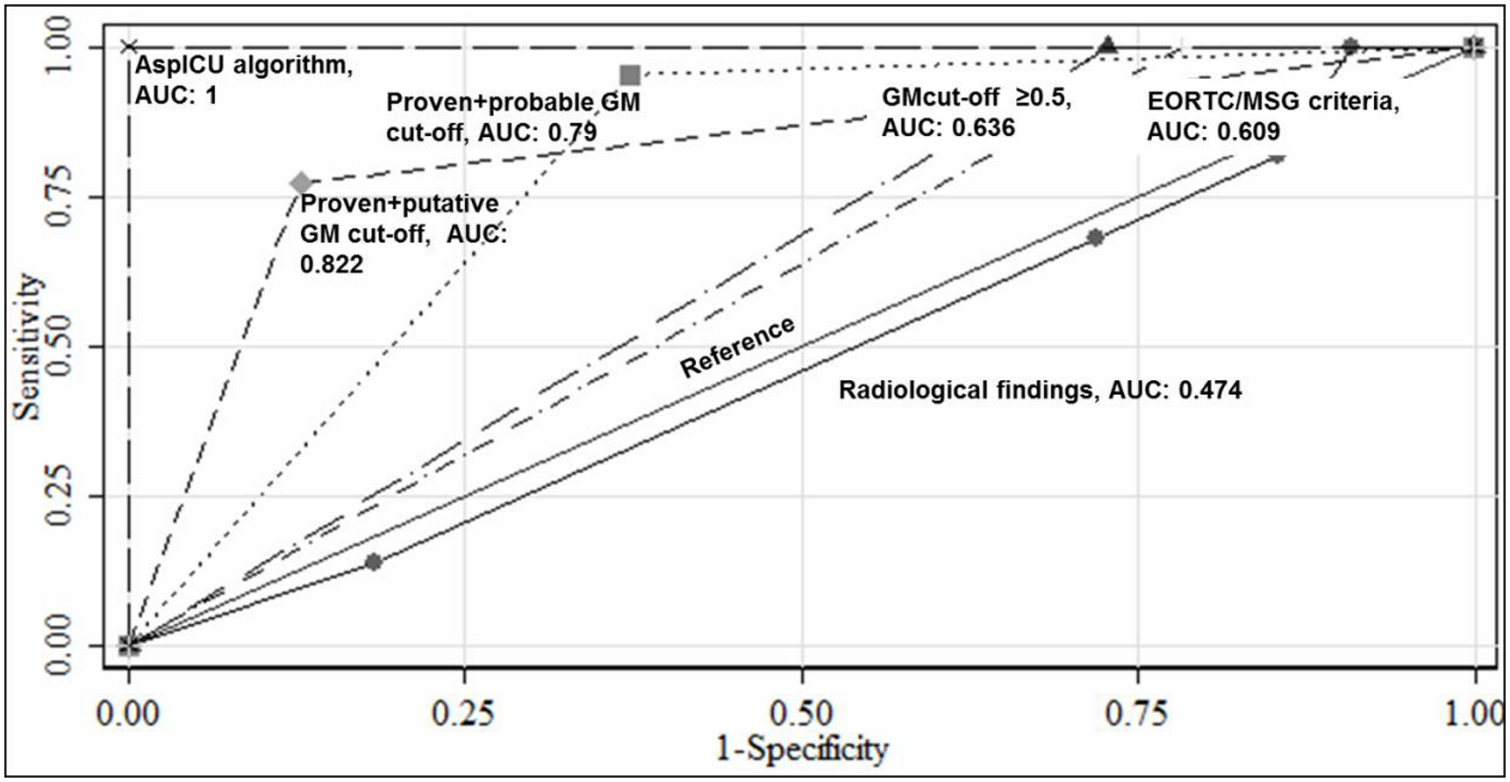
Diagnostic accuracy for the detection of invasive aspergillosis with different tests and diagnostic criteria (using clinically relevant microscopy and culture positive cases as the reference).

The diagnostic accuracy for IA was calculated by AUCs using the GM ODs, radiological findings and two diagnostic criteria. The best predictor of IA was found to be AspICU algorithm (AUC, 1) followed by GM cut-off based on AspICU algorithm (AUC, 0.822) then GM cut-off based on EORTC/MSG criteria (AUC, 0.790) then the standard ≥0.5 GM cut-off (AUC, 0.636) and the lowest was for EORTC/MSG criteria (AUC, 0.609) as radiological findings failed as a marker to predict the infection (AUC, 0.474) (Fig 3).

Galactomannan antigen was detected in serum before radiological findings in 18/22 putative cases with a median duration of 1–2 weeks and in 31/201 No IA cases with a median duration of one week as per the AspICU algorithm classification. Following the EORTC/MSG criteria classification, the GM was detected before radiological findings only in 49/108 probable cases with a median duration of 1 week.

#### Prognostic value of GM assay

The delta (Δ) GM was calculated for all the patients. For 41/235 (17.5%) patients without any antifungal administration, an increase in the GM values was noted on the subsequent samples (Δ GM OD mean: -0.73 and median: -0.53). However, the decrease in GM value was noted with different antifungals “Table 4”.

**Table 4:** Decrease in GM values with decrease duration on different antifungal administration.

	Total no. of patients (N = 235)	Δ GM OD (GM1-GM2) Mean; median	Decrease duration (median) (in days)
No antifungal	41 (17.4%)	-0.73; -0.53	0
Voriconazole	78 (33.2%)	0.63; 0.5	5
Amphotericin B	69 (29.4%)	0.65; 0.21	7
Amphotericin B+ voriconazole	16 (4.3%)	0.6; 0.45	5
Caspofungin	10 (6.8%)	0.26; 0.28	7
Others/Other combinations	21 (8.9%)	0.36; 0.23	12

#### False positivity with piperacillin/tazobactam

Piperacillin/tazobactam therapy was given to 74/235 (31.5%) patients. The median GM OD of the samples drawn from piperacillin/tazobactam non-recipients (161/235, 68.5%) was lower than that of the recipients, median GM = 0.72 (range: 0.21–6.6) vs. 0.78 (range: 0.23–6), respectively. However, no statistical significance was found when positive GM values (≥0.5) of piperacillin/tazobactam recipients (61/74, 82.4%) were analysed vs. non-recipients (117/161, 72.7%) (*p* = 0.105).

### Isolation of *Aspergillus* species and its correlation with GM OD, radiological findings and antifungal administration

From one patient we recovered *Aspergillus nidulans* on repeat isolation from blood culture. The direct microscopy and culture were positive for nine sputum, seven BAL, five endotracheal aspirate (ETA) and one pleural fluid. The most commonly isolated species were *Aspergillus flavus* and *Aspergillus fumigatus* [15 each (9 clinical and 6 colonisation)]. Other species isolated included two *Aspergillus terreus* and one *Aspergillus niger*.

Galactomannan antigen tested positive before radiology w.r.t. *Aspergillus* sp. isolation only in clinical and not in colonisation cases. It was detected earlier in 8/9 *A*.*flavus* isolation cases with median duration of 1–2 week, in 6/9 *A*.*fumigatus* isolation cases with median duration of one week, in the sole case of *A*.*niger* isolation with median duration of 1–2 week and in one of the two *A*.*terreus* isolation cases with median duration of one week. Galactomannan OD increased in cases of *Aspergillus* sp. isolation without any antifungal administration and decreased in cases who received any class of antifungal agents “S1 Table”.

There were 70/235 (29.8%) patients who expired within the 30 days of enrolment in the study. *Aspergillus* culture positivity (34/235, 14.5%) was associated with very high mortality (27/34, 79.4%), (*p*<0.001) “Table 5”. A significant number of non-survivors were colonised patients (12/34, 35.3%) with an unacceptable high mortality (11/12, 91.6%) in the subgroup.

**Table 5:** Mortality in *Aspergillus* culture positive cases.

	Clinical cases (n = 18) (*p*<0.01)			Colonization cases (n = 16) (*p*<0.01)		
	Total	Expired	Survived	Total	Expired	Survived
*A*. *flavus* (15)	9	7 (77.8%)	2 (22.2%)	6	5 (83.3%)	1 (16.7%)
*A*. *fumigatus* (15)	9	6 (66.7%)	3 (33.3%)	6	(100%)	0
*A*. *niger* (1)	1	0	1 (100%)			
*A*. *terreus* (2)	2	2 (100%)	0			
*A*. *nidulans* (1)	1	1 (100%)	0			

**Fig 4:**
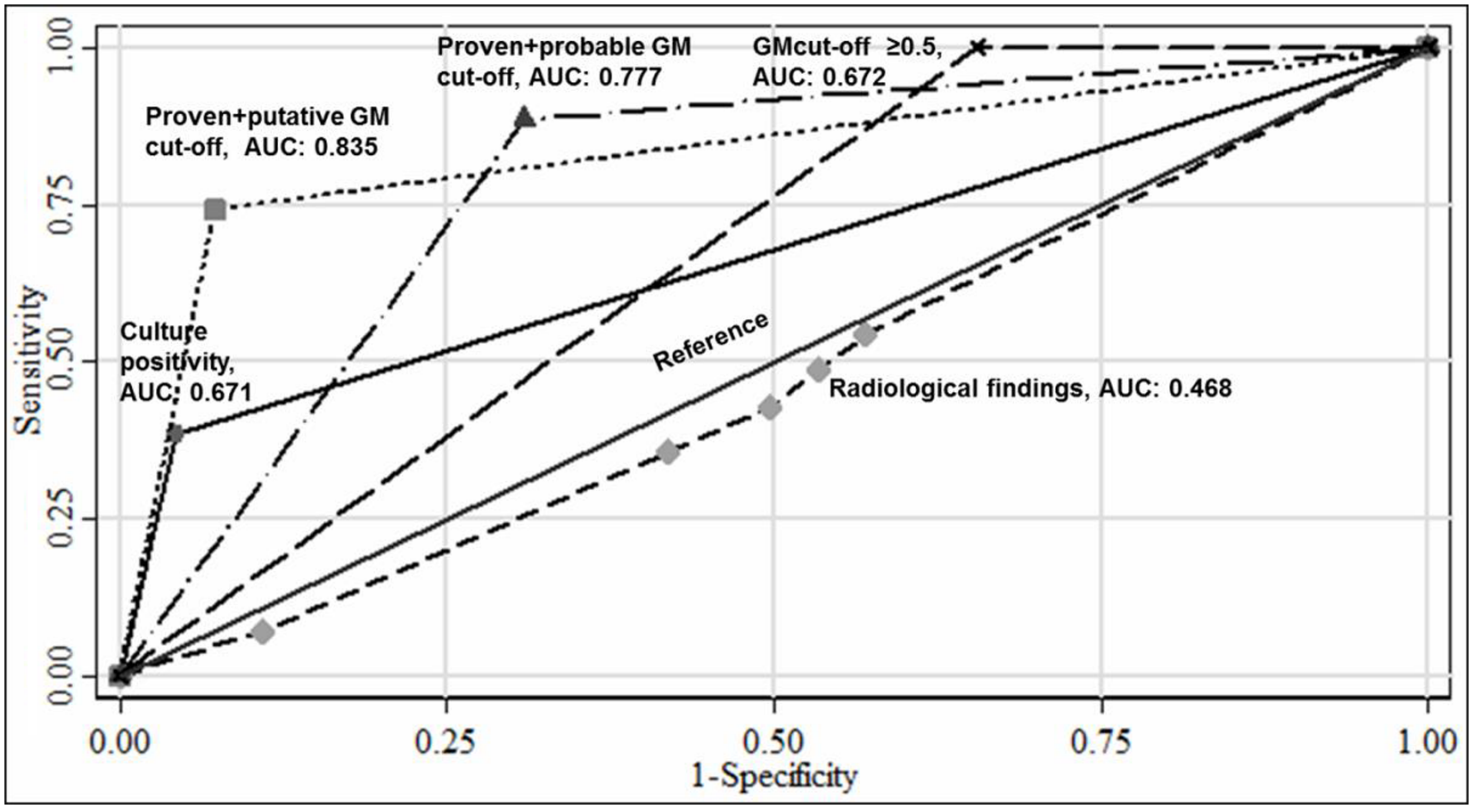
Predictor markers of mortality (using the final outcome as the reference).

The overall culture positivity was a significant independent marker to predict mortality (OR, 14.17; 95% CI, 5.77–34.75). The best predictor for mortality was evaluated among the discriminatory abilities of different GM cut-offs calculated in the study according to the two diagnostic algorithms, culture positivity and radiological findings (Fig 4).

## Discussion

The incidence of IA in ICUs is mostly underestimated, owing to the poor sensitivity and specificity of diagnostic tests (including clinical and radiological findings) and the other difficulties such as coagulation anomalies, difficult oxygenation and LAR consent [10, 21]. In the current study, we analysed the epidemiology of IA in our medicine ICUs. In this study, to improve the case identification rate, an algorithm from IA suspicion from the specifically trained physicians to collection of relevant samples by the highly trained nursing staff, and performance of the tests by mycologists was followed. The reported prevalence of IA in ICUs ranges from 5–7% whereas we observed a higher prevalence of 9.4% (CI: 5.61–13.11) in our patients [2, 6, 9, 22]. There was a large no. of IA patients without any classical cause of immunosuppression 13/22 (59%) which is generally the case in ICU settings [3, 4].

The patients were classified by the two diagnostic algorithms. However, as an indicator of IA, AspICU algorithm with the maximum AUC of 1 superseded EORTC/MSG criteria (AUC: 0.609) (Fig 3). There are few studies where both the algorithms are employed [2, 22, 23]. Most of the available data is retrospective and reports the usefulness of AspICU algorithm over EORTC/MSG criteria’s extrapolations in ICU settings, which is in accordance with our findings [2, 23]. Recently, Schroeder *et al*., evaluated the AspICU algorithm prospectively in critically ill culture positive and negative patients both [23]. The authors suggested inclusion of BAL GM as a modified AspICU algorithm to increase the disease detection rate. However, in another recent study by van Paassen J *et al*., the detailed breakdown of different patient’s condition highlighted the inconclusiveness of both the criteria in clinical practice [22]. It may therefore be a better approach to follow a criterion with modifications better suited locally with flexibility of one’s own clinical settings. The conditions vary from place to place, for example, the ICU stay in our hospital is median 3 days when compared to the western data of ≥ 12 days of ICU hospitalisation [24].

The interdependent risk factors found on multivariate analysis following both the diagnostic criteria in this study were a mix of few classical and many non-classical risk factors which is in accordance with the existing literature “Table 2” [3].

Radiology including CT scans in ICU settings is often not possible due to the critical conditions of the patients. However, it was performed on ~80% of our patients. The imaging findings were only found independently significant in this study (unadjusted ORs, >1). The classical halo sign mentioned as an inclusion factor in the EORTC/MSG criteria was not observed but the patients who had new pulmonary infiltrates or progressive consolidation on CXR/CT scan (new, combinational or progressive findings) were included as a modification of existing criteria based on the reported non- specific radiological signs in ICU patients [2, 3, 6].

Serial serum samples were obtained from all the patients whereas BAL was only obtained from 24/235 (10.2%), probably because of the unstable vitals (heart rate, blood pressure etc.) of our ICU patients. Galactomannan antigen tested positive (≥0.5) for 178 of 235 (75.1%) patients. The sensitivity and specificity of the GM assay were found higher following the AspICU algorithm than the EORTC/MSG criteria. The significant GM cut-offs for the AspICU algorithm (GM cut-off, ≥1.24; AUC, 0.843) showed better discrimination vs. EORTC/MSG criteria (GM cut-off, ≥0.78; AUC, 0.614). However, with the inclusion of standard GM cut-off (≥0.5), the proportion of proven/putative IA cases in culture negative cases changed (34/34, 100% if positive cultures vs. 144/201, 71.6%) if negative cultures). In our clinical settings with a large heterogeneous population, therefore, to ignore GM testing can render an increased likelihood of a large number of patients left untreated.

In this study, 37.1% (49/132) patients were positive for galactomannan antigen before appearance of radiological signs, which is similar to the published literature in acute leukemia and hematopoietic stem cell transplantation patients [25]. The prognostic value of the GM test was also statistically analysed using bonferroni’s and bartlett’s test for equal variances. The decrease in GM OD was not statistically significant among the group of different antifungals administered, however, the increase in subsequent GM OD was statistically significant between the patient group of no antifungal and antifungals administered (*p*<0.001). A similar mean decrease in GM OD was seen (~0.6) with voriconazole and amphotericin B administration whereas there were considerable difference in the median GM OD and duration decrease with the two antifungals “Table 4”. Similar findings were reported by Chai LYA *et*. *al*. with only difference of using conventional amphotericin B in their study as compared to the liposomal formulations used in ours [26].

The false positivity with piperacillin/tazobactam in GM assay for IA detection has been explored extensively, for a long period the drug combination was thought to be responsible for false positives [25, 27, 28]. However, in this study, GM positivity was independent of piperacillin/tazobactam therapy (*p* = 0.105) similar to recently published data [29–33].

In this study, the calculated putative and probable GM cut-offs were good markers to predict mortality (AUCs ≥0.7) (Fig 4). The *Aspergillus* culture positivity was associated with very high mortality (79.4%) and was found to be an independent risk marker for mortality (unadjusted OR, 14.1; 95% CI, 5.77–34.75).

We also analysed the association of different positive GM cut offs described in literature w.r.t. mortality reported in this study “Table 6”.

**Table 6:** Described significant GM cut-offs in literature and the observed mortality in this study.

No. of patients (n) and hypothetical GM cut-offs of:	Survivors (n = 165)	Non survivors (n = 70)	*P* value	Unadjusted Odds ratio (95% CI)
≥0.500 (n = 178)	108 (65.4%)	70 (100%)	0.000	predicts failure perfectly
≥0.8 (n = 114)	51 (30.9%)	63 (90%)	0.000	20.11 (8.61–46.96)
≥1 (n = 84)	23 (13.9%)	61 (87.1%)	0.000	41.84 (18.3–95.67)
≥1.5 (n = 48)	6 (3.6%)	42 (60%)	0.000	39.75 (15.44–102.27)

An increase in GM OD was seen in 40/235 (17%) patients on the second sample and none of them received any antifungals during their ICU stay. There were 26/40 (65%) patients who expired and 14/40 (35%) survived. There was only one patient in whom a decrease in GM OD on the second sample was seen even without administration of any antifungal agent, this may be because before the collection of first sample this patient was on parenteral nutrition. An increase in subsequent serum GM OD was found independently significant for mortality (*p*<0.001) (odds ratio: 6.37; 95% CI: 3.06–13.24). Thereby suggesting ignoring GM monitoring can also have repercussions on patient outcome.

### Limitations

This study had limitations as it was physician driven (based on clinical suspicion of IA) in a cohort of critically ill patients at particular high risk: this might be linked to the high incidence of IA. Other limitations included the relatively small sample size, lack of autopsy and BAL samples.

## Supporting information

S1 Table Decrease in GM OD after antifungal administration in the clinical cases with *Aspergillus* sp. isolation. [Note: N: total number; SD: standard deviation].
(DOCX)
