# Supplementary material for: Serum galactomannan antigen as a prognostic and diagnostic marker for invasive aspergillosis in heterogeneous medicine ICU patient population
Source: PLoS One. 2018 Apr 23;13(4):e0196196. doi: 10.1371/journal.pone.0196196 (PMC5912734; doi:10.1371/journal.pone.0196196)
Supplement: S1 Table — [Note: N: total number; SD: standard deviation]. (DOCX) [file pone.0196196.s001.docx]

S1 Table: Decrease in GM OD after antifungal administration in the clinical cases with *Aspergillus* sp. isolation

|  | No *Aspergillus* | *A. flavus* | *A. fumigatus* | *A. niger* | *A. terreus* | *A. nidulans* |
| --- | --- | --- | --- | --- | --- | --- |
| No antifungal | | | | | | |
| N | 33 | 4 | 3 |  | 1 |  |
| Mean+-SD | -0.66±0.76 | -1.01± 0.58 | -0.59±0.32 |  | -1.16 |  |
| Median | -0.41 | -0.92 | -0.46 |  | -1.16 |  |
| Decrease duration (in days): mean ± SD; median | NA | NA | NA |  | NA |  |
| Voriconazole | | | | | | |
| N | 71 | 2 | 4 |  |  | 1 |
| Mean+-SD | 0.62±0.45 | 0.49±0.53 | 0.76±0.3 |  |  | 0.79 |
| Median | 0.49 | 0.49 | 0.78 |  |  | 0.79 |
| Decrease duration (in days): mean ± SD; median | 4.98±1.44; 5 | 3±1.41; 3 | 5±0.81; 5 |  |  | 5 |
| Amphotericin B | | | | | | |
| N | 66 |  | 2 |  | 1 |  |
| Mean+-SD | 0.64±1.34 |  | 1.14±1.2 |  | 0.6 |  |
| Median | 0.2 |  | 1.14 |  | 0.6 |  |
| Decrease duration (in days): mean ± SD; median | 10.56±4.4; 7 |  | 7±0; 7 |  | 7; 7 |  |
| Caspofungin | | | | | | |
| N | 10 |  |  |  |  |  |
| Mean+-SD | 0.26±0.13 |  |  |  |  |  |
| Median | 0.28 |  |  |  |  |  |
| Decrease duration (in days): mean ± SD; median | 6.6±2.41; 7 |  |  |  |  |  |
| Voriconazole + Amphotericin B | | | | | | |
| N | 14 | 1 |  | 1 |  |  |
| Mean+-SD | 0.47±0.26 | 2.55 |  | 0.46 |  |  |
| Median | 0.42 | 2.55 |  | 0.46 |  |  |
| Decrease duration (in days): mean ± SD; median | 5.21±1.57; 5.5 | 3; 3 |  | 4; 4 |  |  |
| Others/ other antifungal combinations | | | | | | |
| N | 19 | 2 |  |  |  |  |
| Mean+-SD | 0.25±0.16 | 1.43±0.87 |  |  |  |  |
| Median | 0.22 | 1.43 |  |  |  |  |
| Decrease duration (in days): mean ± SD; median | 12.36±3.89; 12 | 6±1.41; 6 |  |  |  |  |

[Note: N: total number; SD: standard deviation]
